# Oncological results in rectal cancer patients with a subcentimetre distal margin after laparoscopic‐assisted sphincter‐preserving surgery

**DOI:** 10.1111/ans.17503

**Published:** 2022-01-27

**Authors:** Chenghai Zhang, Ming Cui, Jiadi Xing, Hong Yang, Xiangqian Su

**Affiliations:** ^1^ Key Laboratory of Carcinogenesis and Translational Research (Ministry of Education), Department of Gastrointestinal Surgery IV Peking University Cancer Hospital and Institute Beijing China

**Keywords:** distal resection margin, local recurrence, overall survival, rectal cancer, sphincter‐preserving surgery

## Abstract

**Background:**

Distal resection margin (DRM) is closely associated with sphincter‐preserving surgery and oncological safety for patients with mid‐low rectal cancers. However, the optimal DRM has not been determined.

**Methods:**

Data of 378 rectal cancer patients who underwent laparoscopic‐assisted sphincter‐preserving surgery from 2009 to 2015 were retrospectively analysed. Patients were divided into two groups based on DRM: ≤1 cm (*n* = 74) and >1 cm (*n* = 304). To minimize the differences between the two groups, propensity‐score matching on baseline features was performed.

**Results:**

Before propensity‐score matching, no significant differences in 5‐year disease‐free survival (DFS) (92.8% versus 81.3%, *P* = 0.128) and 5‐year overall survival (OS) (83.7% versus 82.2%, *P* = 0.892) were observed in patients with DRMs of ≤1 cm (*n* = 74) and >1 cm (*n* = 304), respectively. After propensity‐score matching (1:1), there were also no significant differences in DFS (88.1% versus 78.2%, *P* = 0.162) and OS (84.5% versus 84.9%, *P* = 0.420) between the DRM of ≤1 cm group (*n* = 65) and >1 cm group (*n* = 65), respectively. A total of 44 patients received preoperative chemoradiotherapy (CRT). In this cohort, the 5‐year local recurrence (LR) rates (*P* = 0.118) and the 5‐year DFS rates (*P* = 0.298) were not significantly different between the two groups. A total of 334 patients received surgery without neoadjuvant CRT. There were also no significant differences in the 5‐year LR rates (*P* = 0.150) and 5‐year DFS rates (*P* = 0.172) between the two groups.

**Conclusions:**

When aiming to achieve at least a 1–2 cm distal clinical resection margin, a histological resection margin of <1 cm on the DRM gave equivalent clinical outcomes to a DRM of >1 cm.

## Graphical abstract

The optimal distal resection margin (DRM) has been debated in sphincter‐saving surgery for patients with low‐lying rectal cancers. We analysed the impact of a DRM of 1 cm on local recurrence and long‐term survival for patients who underwent sphincter‐preserving surgery. No matter whether patients received neoadjuvant therapy, a DRM of 1 cm may be acceptable.

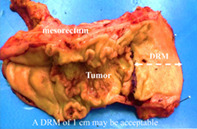

## Introduction

Circumferential resection margin (CRM) and distal resection margin (DRM) are strongly associated with local recurrence (LR) and distant metastasis. Positive distal margins are associated with worse oncological results and chemoradiotherapy (CRT) cannot compensate for this.^1^, ^2^ Therefore, a DRM of at least 5 cm was suggested for patients with locally advanced rectal cancers in the past.^3^, ^4^ However, with the advent of total mesorectal excision (TME) surgery,^5^ neoadjuvant CRT (NCRT)^6^, ^7^, ^8^ and advances in laparoscopic surgery,^9^ shorter DRM was found to be oncologically adequate.^10^, ^11^

However, the optimal DRM is still controversial in sphincter‐saving surgery. Several studies showed that a DRM of less than 1 cm did not jeopardize the long‐term survival and LR.^11^, ^12^, ^13^ Nevertheless, Kondo *et al*. revealed that a DRM of 2 cm was required for patients with low‐lying rectal cancer even if they were offered NCRT.^14^ So, the optimal DRM and its oncological implications for rectal carcinoma patients with or without NCRT need further investigation.

## Methods

### Patients

Clinicopathological data of 378 consecutive patients with rectal cancers treated by laparoscopy‐assisted anterior resection with standard TME were collected from January 2009 through December 2015. Patients with a history of other malignant tumours, distant metastasis or a pathologically proven positive CRM or DRM were excluded. Clinicopathological data were obtained through electronic medical records, and survival data through the special follow‐up centre of our hospital. Informed consent was obtained from each patient enrolled in the study. All operations performed in this study involving patients were in line with the ethical standards of the Ethics Committee of Peking University Cancer Hospital & Institute.

### NCRT and surgical procedure

NCRT was recommended for participants with cT3 or T4 tumour and/or lymph nodes metastasis. Three‐dimensional conformal intensity‐modulated radiotherapy was used. NCRT protocol comprised a total irradiation dose of 50.4 Gy, delivered in 2 Gy fractions, 5 days per week for 5 weeks. Concurrent chemotherapy consisted of continuous infusion of 5‐fluorouracil and leucovorin or oral capecitabine.

Laparoscopy‐assisted low anterior resection was performed by experienced surgeons. For lesions close to the anorectal junction, when the aim of a DRM greater than 1 cm cannot be achieved, a microscopically negative DRM was acceptable. The DRM of the fresh resected specimen was inspected by the surgeon. If it was suspiciously positive or microscopically involved at a frozen‐section examination, an additional part of the distal rectum was removed or abdominoperineal resection was adopted. Digestive tract reconstruction was performed using double‐stapled or hand‐sewn anastomosis.

### Histopathology

The DRM was measured following the microscopic examination of the formalin‐fixed specimen. It was defined as the closest distance from the lowest border of the lesion (or the scar tissue after NCRT) to the distal mucosal resection margin. The doughnut was examined microscopically, but not included in this measurement.

### Definition of recurrence

LR, including anastomotic and pelvic lymph nodes recurrence, was defined as any clinically or histopathologically confirmed carcinoma recurrence after primary operation. Distant metastasis is defined as the metastasis of cancer cells to distant organs (e.g. lung, liver, bone) or lymph nodes (e.g. para‐aortic or supraclavicular lymph nodes).

### Statistical analysis

Categorical variables are presented as frequencies and percentages. Continuous variables are described as mean ± standard deviation (SD) or as median for skewed or kurtotic distributions. Pearson's chi‐squared test was used to determine differences between categorical variables. Student's *t*‐test and Mann–Whitney *U*‐test were used to analyse continuous variables. Kaplan–Meier method with the log‐rank test was used for survival analysis. A *P*‐value of <0.05 was considered statistically significant.

Propensity‐score matching was performed to minimize baseline differences. Nearest‐neighbour matching without replacement was used. Matching was conducted with the use of a 1:1 matching protocol, with a calliper width equal to 0.2 of the SD of the logit of the propensity score. All analyses were carried out with SPSS version 23.0 (SPSS Inc., Chicago, IL, USA).

## Results

### Clinicopathological characteristics of patients with mid‐low rectal cancer

Detailed clinicopathological data of the 378 rectal cancer patients are shown in Table 1. The mean DRM length was 0.8 ± 0.3 cm in the DRM of ≤1 cm group and 2.6 ± 1.0 cm in the DRM of >1 cm group. The mean distance from the anal verge in the DRM of ≤1 cm group was significantly different from that in the DRM of >1 cm group (8.3 ± 3.3 versus 9.3 ± 2.8 cm, *P* = 0.017). The groups were comparable in gender, age, preoperative CRT, tumour differentiation and lymphovascular invasion. There were more patients with pT1/T2 in the DRM of ≤1 cm group (48.7% versus 21.0%, *P* < 0.001), as well as more TNM p‐stage I/II (62.2% versus 53.9%, *P* < 0.001), respectively.

**Table 1:** Features of the patients in different subgroups

Variables	Before matching			After matching		
	DRM ≤1 cm (*n* = 74)	DRM >1 cm (*n* = 304)	*P*	DRM ≤1 cm (*n* = 65)	DRM >1 cm (*n* = 65)	*P*
Distal margin (cm), (mean ± SD)	0.8 ± 0.3	2.6 ± 1.0		0.8 ± 0.3	2.4 ± 0.9	
Age (mean ± SD)	63 ± 10.9	60 ± 10.3	0.055	63.2 ± 11.1	63.9 ± 8.9	0.709
Sex			0.932			0.598
Male	40	166		33	36	
Female	34	138		32	29	
Tumour distance from AV (cm, range)	8.3 ± 3.3	9.3 ± 2.8	0.017	8.5 ± 3.4	8.5 ± 2.5	1.000
Pathological T stage			<0.001			0.932
T1	12	18		8	6	
T2	24	46		21	20	
T3	36	188		34	37	
T4	2	52		2	2	
TNM stage			<0.001			0.519
I	28	50		23	17	
II	18	114		18	20	
III	28	140		24	28	
Preoperative CRT			0.171			0.435
Yes	12	32		10	7	
No	62	272		55	58	
Tumour differentiation			0.745			0.739
Well	4	20		4	3	
Moderately	56	242		49	54	
Poorly	12	36		10	7	
Uncertainly	2	6		2	1	
Perineural invasion, *n* (%)	4 (5.4)	16 (5.3)	1.000	2 (3.1)	7 (10.8)	0.167
Lymphovascular invasion, *n* (%)	8 (10.8)	44 (14.5)	0.412	8 (12.3)	8 (12.3)	1.000

Data are given as number of patients with percentage.

AV, anal verge; CRT, chemoradiotherapy; DRM, distal resection margin.

After applying propensity‐score matching strategy (1:1), 65 patients with a DRM of ≤1 cm were matched to 65 patients with a DRM of >1 cm. Based on the results presented in Table 1, there were no significant differences in baseline clinicopathological data between the two groups (*P* > 0.05).

### Oncological results in relation to the different distal margins regardless of NCRT

Before propensity‐score matching, five out of 74 (6.8%) patients with a DRM of ≤1 cm developed LR, and 18 out of 304 (5.9%) patients with a DRM of >1 cm had LR (*P* = 0.920). The distant metastasis rate was similar between the two groups (8.1% versus 13.2%, *P* = 0.183). After propensity‐score matching (Table 2), the LR rate was same in both groups (7.7% versus 7.7%, *P* = 1.000). No significant difference was observed in distant metastasis between the two groups (9.2% versus 15.4%, *P* = 0.286).

**Table 2:** Oncological results in relation to different distal margins

	Before matching			After matching		
Variable	DRM ≤1 cm	DRM >1 cm	*P*	DRM ≤1 cm	DRM >1 cm	*P*
	(*n* = 74)	(*n* = 304)		(*n* = 65)	(*n* = 65)	
Median follow‐up, months (range)	78 (3–126)	70 (8–132)	0.646	79 (3–126)	65 (15–118)	0.122
Local recurrence (%)	5/74 (6.8)	18/304 (5.9)	0.92	5/65 (7.7)	5/65 (7.7)	1.000
Metastasis (%)	6/74 (8.1)	40/304 (13.2)	0.183	6/65 (9.2)	10/65 (15.4)	0.286
5‐year DFS (%)	92.8	81.3	0.128	88.1	78.2	0.162
5‐year OS (%)	83.7	82.2	0.892	84.5	84.9	0.420

Data are given as number of patients with recurrence/total number of patients.

DFS, disease‐free survival; DRM, distal resection margin; OS, overall survival.

The patterns of LR and distant metastasis are presented in Table 3. With regard to LR, pelvic lymph node recurrence was more common than anastomotic recurrence in both groups. These five patients with LR were randomly distributed in the DRM of ≤1 cm group. Detailed information is shown in Table 4 and Figure S1. With regard to distant metastasis, the lung was the most common metastatic organ in the two groups. The specific distant metastatic locations are shown in Table 3.

**Table 3:** Patterns of LR and distant metastasis in the subgroups

Recurrence sites	DRM ≤1 cm (*n* = 74)	DRM >1 cm (*n* = 304)
LR, *n* (%)		
Anastomotic	2 (2.7)	6 (2.0)
Pelvic LN	3 (4.1)	12 (3.9)
DM, *n* (%)		
Liver	0 (0.0)	12 (3.9)
Lung	4 (5.4)	16 (5.3)
Liver and lung	2 (2.7)	8 (2.6)
Para‐aortic lymph nodes	1 (1.4)	2 (0.7)
Ovary	0 (0.0)	2 (0.7)

DM, distant metastasis; DRM, distal resection margin; LN, lymph node; LR, local recurrence.

**Table 4:** More information about LR in the DRM of ≤1 cm group

Case number	Sex (M/F)	Preoperative CRT	DRM, mm	pTNM	DFS, months	Sites of LR
1	F	No	10	pT3N1M0	32	Anastomosis
2	M	No	10	pT1N0M0	61	Pelvic lymph nodes
3	M	No	4	pT3N0M0	25	Anastomosis
4	M	Yes	5	PCR	10	Pelvic lymph nodes
5	F	No	6	pT2N1M0	40	Pelvic lymph nodes

CRT, chemoradiotherapy; DFS, disease‐free survival; DRM, distal resection margin; F, female; LR, local recurrence; M, male; PCR, pathological complete remission.

**Fig. 1:**
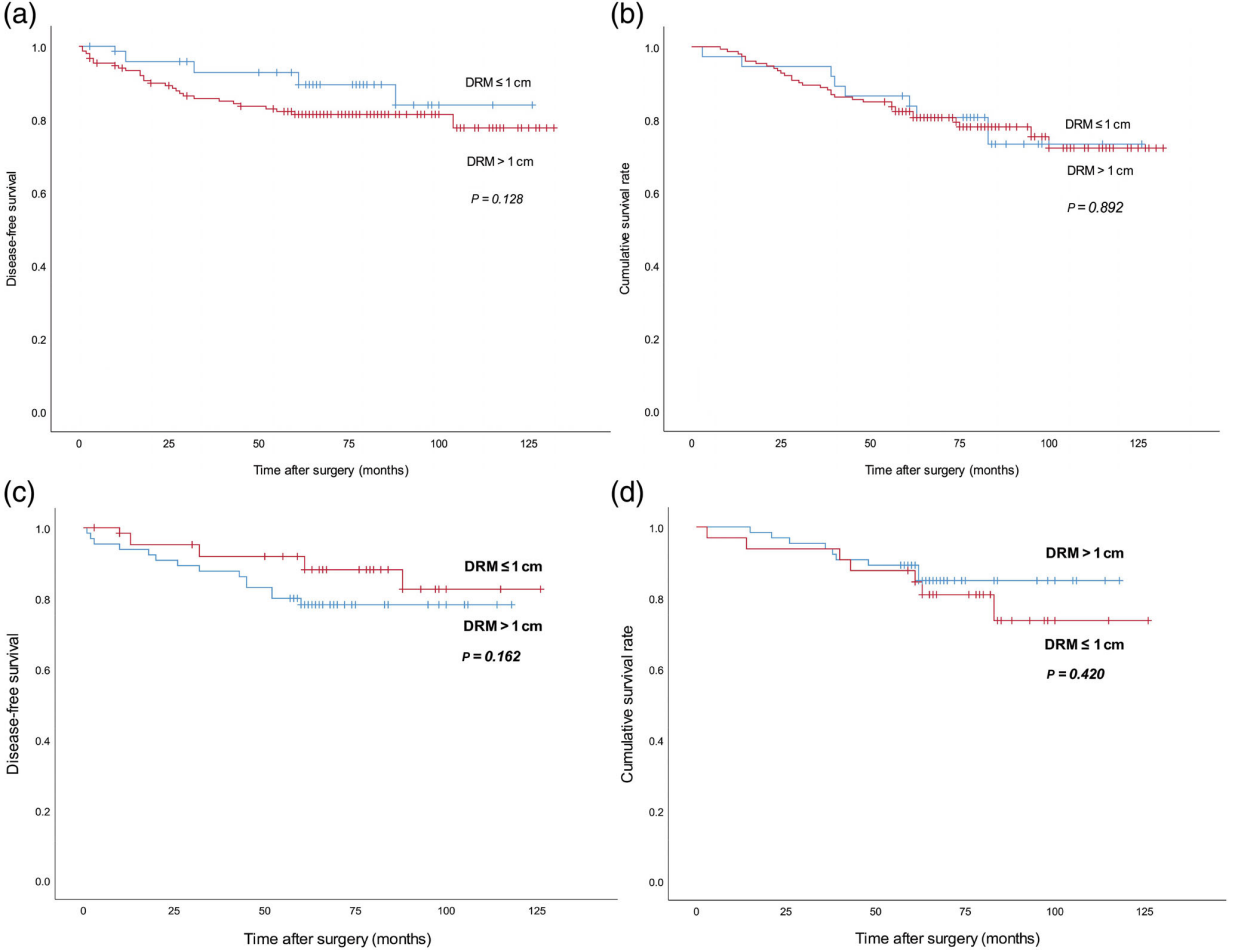
Overall survival (OS) and disease‐free survival (DFS) related to the length of the DRM. Before propensity‐score matching, no significant differences in the 5‐year DFS (a) and 5‐year OS (b) were observed in the DRM of ≤1 and >1 cm groups. After propensity‐score matching, there were also no significant differences in the 5‐year DFS (c) and 5‐year OS d between the DRM of ≤1 and >1 cm groups. DRM, distal resection margin.

Before propensity‐score matching, the disease‐free survival (DFS) rate was 92.8% in patients with a DRM of ≤1 cm group and 81.3% in patients with a DRM of >1 cm group (*P* = 0.128). The overall survival (OS) rate at 5 years was 83.7% in the DRM of ≤1 cm group and 82.2% in the DRM of >1 cm group (*P* = 0.892; Fig. 1). After propensity‐score matching, there were also no significant differences in DFS (88.1% versus 78.2%, *P* = 0.162) and OS (84.5% versus 84.9%, *P* = 0.420) between the DRM of ≤1 cm group (*n* = 65) and the DRM of >1 cm group (*n* = 65), respectively (Fig. 1).

### Subgroup analysis of OS stratified by DRM and the use of NCRT

**Fig. 2:**
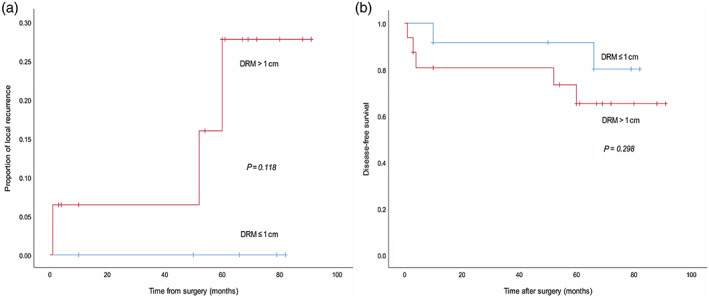
Local recurrence (LR) and disease‐free survival (DFS) of patients with neoadjuvant chemoradiation (stratified by distal margin of ≤1 cm). No significant differences in LR (a) and 5‐year DFS (b) were observed in the DRM of ≤1 and >1 cm groups. DRM, distal resection margin.

A total of 44 patients received preoperative CRT. In this cohort, the 5‐year LR rates were similar between the DRM of ≤1 cm group and the DRM of >1 cm group (8.3% versus 1.9%, *P* = 0.118). The estimated 5‐year DFS rate was not significantly different between the two groups (83.3% versus 68.8%, *P* = 0.298; Table 5, Fig. 2).

**Table 5:** Kaplan–Meier estimates of 5‐year LR and 5‐year DFS stratified by DRM and NCRT

	Variable	Group (*n*)	No. of events (%)	*P* (log‐rank)
NCRT (*n* = 44)	LR	DRM ≤1 cm (12)	1 (8.3)	0.118
		DRM >1 cm (32)	6 (1.9)	
	DFS	DRM ≤1 cm (12)	10 (83.3)	0.298
		DRM >1 cm (32)	22 (68.8)	
Surgery alone (*n* = 334)	LR	DRM ≤1 cm (62)	0 (0.0)	0.150
		DRM >1 cm (272)	10 (3.7)	
	DFS	DRM ≤1 cm (62)	55 (93.5)	0.172
		DRM >1 cm (272)	226 (83.8)	

DFS, disease‐free survival; DRM, distal resection margin; LR, local recurrence; NCRT, neoadjuvant chemoradiotherapy.

**Fig. 3:**
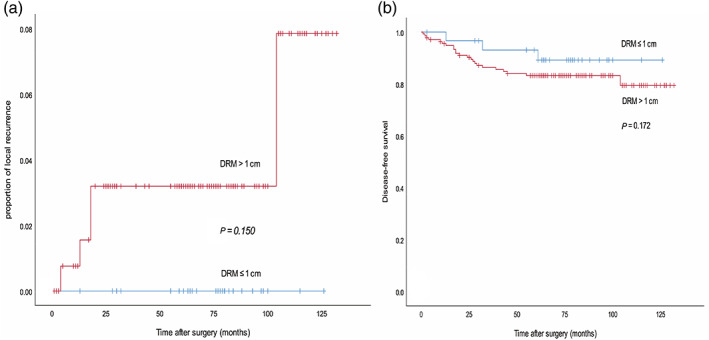
Local recurrence (LR) and disease‐free survival (DFS) of patients without neoadjuvant chemoradiation (stratified by distal margin of ≤1 cm). No significant differences in LR (a) and 5‐year DFS (b) were observed in the DRM of ≤1 and >1 cm groups. DRM, distal resection margin.

Three hundred and thirty‐four patients received surgery alone without NCRT. Consistent with the neoadjuvant chemoradiation group, there were also no significant differences in LR rate (0% versus 3.7%, *P* = 0.150) and DFS (93.5% versus 83.8%, *P* = 0.172; Table 5, Fig. 3) between the DRM of ≤1 cm group (*n* = 62) and the DRM of >1 cm group (*n* = 272), respectively.

## Discussion

The optimal length of DRM has not been determined during sphincter‐sparing surgery for patients with mid‐low rectal cancers due to the lack of evidence from high‐level randomized controlled studies. Therefore, the investigation of the effect of the DRM on the oncological results of the low‐lying rectal cancer patients is necessary and of important clinical significance.

The length of the DRM is mainly dependent on the tumour location in the rectum. In this study, the mean distance from the anal verge in the DRM of ≤1 cm group was shorter than that in the DRM of >1 cm group, similar to previous studies.^11^, ^15^, ^16^ Meanwhile, we found that the proportion of patients with T1 and T2 stages in the DRM of ≤1 cm group was higher than that in the control group. Similarly, the proportion of patients with stage I and II in the DRM of ≤1 cm group was higher than that in the DRM of >1 cm group. This is because of case selection. This would favour a lower LR and distant metastasis rate in the DRM of ≤1 cm group, which was one of the reasons for performing propensity matching.

Overall, there were no significant differences in the 5‐year LR rate, 5‐year DFS and 5‐year OS between the two groups in this study. Similarly, a systemic review of 5574 rectal cancer patients conducted by Bujko *et al*. evaluated whether a DRM of <1 cm jeopardizes oncological safety.^17^ They concluded that a DRM of <1 cm did not compromise oncological outcomes. Kang *et al*. reported that the 5‐year LR rate was 8.8% in the DRM of ≤1 cm group and 8.5% in the DRM of >1 cm group (*P* = 0.630).^16^ Admittedly, the proportion of patients with a DRM of ≤5 mm in the DRM of ≤1 cm group is relatively low (25.7%, 19/74) in the present study, which may affect the results to a certain extent. We will further compare the survival differences between patients with a DRM of ≤5 mm and patients with a DRM of 5–10 mm.

Many studies attempted to define the narrowest sufficient DRM (5 mm, 8 mm, 1 cm and 2 cm) in patients with sphincter‐preserving surgery.^11^, ^12^, ^13^, ^14^, ^15^, ^18^, ^19^ However, analysis of the impact of DRM on survival results was not well stratified by NCRT. A subgroup analysis of this study showed that there were no significant differences in the 5‐year LR and 5‐year DFS between the two groups. Similarly, Manegold *et al*. found that the 5‐year LR rate was 6.7% in patients with a DRM of <1 cm and 5.5% in patients with a DRM of ≥1 cm.^11^

The effect of NCRT or preoperative chemotherapy on the degree of shrinkage and regression of the primary tumour may be different. Many studies showed that distal intramural spread greater than 1 cm was only in 0–5% of patients receiving NCRT.^20^, ^21^, ^22^ However, Kondo *et al*. analysed 71 patients with low rectal carcinoma who received preoperative chemotherapy.^14^ They found that 42 (59%) patients had distal spread. Distal spreads of 1–9 mm, 10–19 mm and ≥2 cm were observed in 27 (38%), 11 (15%) and four (6%) patients, respectively. The results of Kondo *et al*.'s study were significantly different from those of previous studies and the present study, mainly because their patients only received chemotherapy without radiotherapy.^20^, ^21^, ^22^ The specific mechanism of the difference between preoperative CRT and preoperative chemotherapy on the distal spread was still unclear. It should be noted that for patients with high‐risk factors who underwent preoperative chemotherapy, a DRM of 1 cm may not be sufficient.

By subgroup analysis, a DRM of ≤1 cm did not compromise the oncological outcomes of patients who received TME surgery alone. The introduction and application of TME principle for advanced rectal cancer have significantly reduced LR and improved a safe DRM.^5^, ^23^, ^24^ A study including 152 mid‐low rectal cancer patients with TME surgery alone reported that there were no significant differences in 10‐year recurrence rates between the DRM of ≤1 cm group and the DRM of >1 cm group (0.0% versus 3.6%, *P* = 0.27).^25^

The present study has several drawbacks. First, data on the DRM distance were extracted from the pathology report and not measured by a fixed pathologist. Theoretically speaking, these data may have errors and cannot be verified repeatedly. Second, although the total sample size is large, the number of patients with a DRM of ≤1 cm group is small. Third, the proportion of patients receiving neoadjuvant therapy was low. On one hand, doctors were more likely to recommend patients with tumour close to CRM or obvious lymph nodes metastasis to receive preoperative radiotherapy. On the other hand, some patients were unwilling to receive NCRT because of heavy financial burden, long treatment cycle and fear of increased post‐operative complications. In addition, not all specimens in this study were pinned. So, the length of DRM may be affected by different measurement methods.

## Conclusions

No matter whether patients with mid‐low rectal cancers received NCRT, there were no significant differences in 5‐year LR rate, 5‐year DFS and 5‐year OS between the DRM of ≤1 cm group and the DRM of >1 cm group. When aiming to achieve at least a 1–2 cm distal clinical resection margin, a histological resection margin of <1 cm on the DRM, ignoring the donut, gave equivalent clinical outcomes to a DRM of >1 cm. Besides, low anterior resection with a subcentimetre DRM was more suitable in patients with low‐risk LR factors such as T2/T3 and negative CRM.

## Conflict of interest

None declared.

## Author contributions

**Chenghai Zhang:** Conceptualization; data curation; formal analysis; methodology; project administration; visualization; writing – original draft; writing – review and editing. **Ming Cui:** Data curation; investigation; supervision. **Jiadi Xing:** Data curation; resources; software; writing – review and editing. **Hong Yang:** Resources; software; supervision; validation. **Xiangqian Su:** Conceptualization; funding acquisition; project administration.

## Supporting information


**Figure S1.** The distribution of patients with local recurrence in the distal resection margin of ≤1 cm group. Red bars represent recurrence cases.
