# Supplementary figures and images for: Oncological results in rectal cancer patients with a subcentimetre distal margin after laparoscopic‐assisted sphincter‐preserving surgery
Source: ANZ J Surg. 2022 Jan 27;92(6):1454–60. doi: 10.1111/ans.17503 (PMC9305552; doi:10.1111/ans.17503)

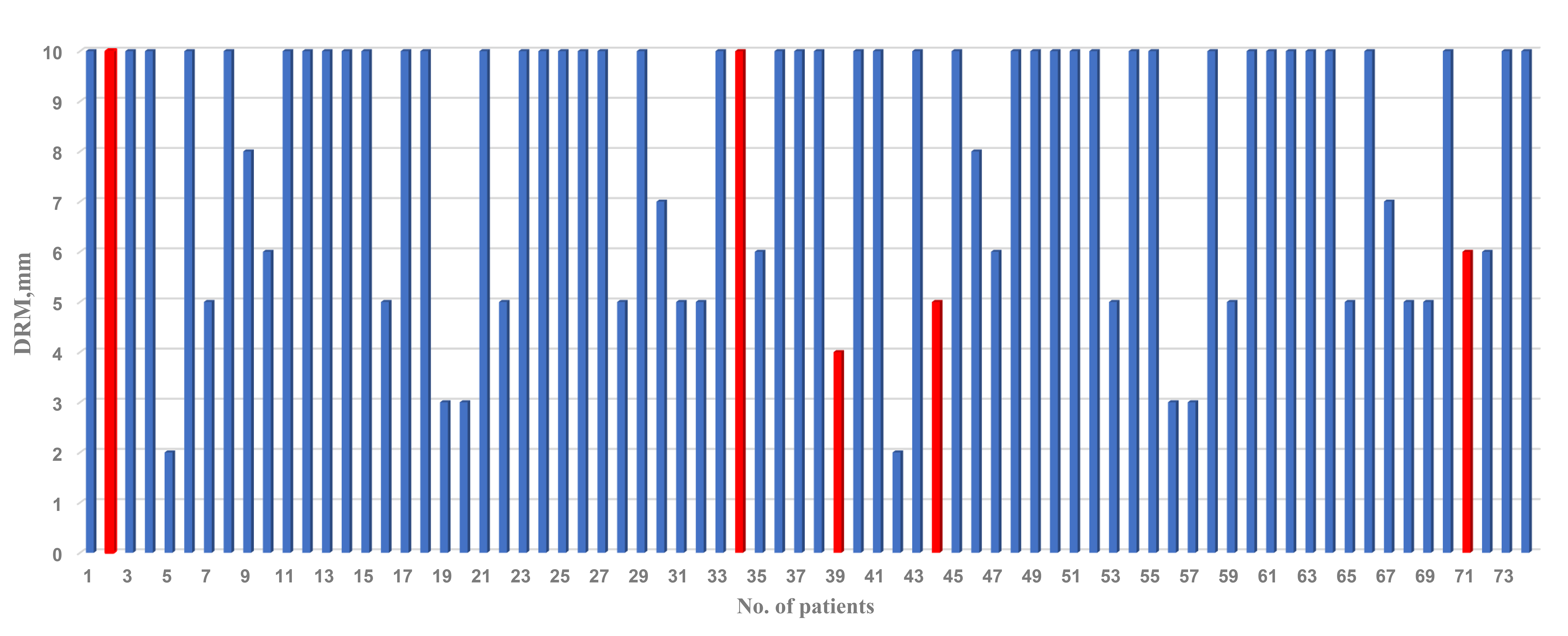

Supplement: Supplementary file 1 — Figure S1. The distribution of patients with local recurrence in the distal resection margin of ≤1 cm group. Red bars represent recurrence cases. [file ANS-92-1454-s001.tif]
